# The internationalization of TCM towards Portuguese-speaking countries

**DOI:** 10.1186/s13020-021-00491-6

**Published:** 2021-08-19

**Authors:** Xiaoting Zheng, Liyang Lyu, Hong Lu, Yuanjia Hu, Ging Chan

**Affiliations:** State Key Laboratory of Quality Research in Chinese Medicine, Institute of Chinese Medical Sciences, University of Macau, Taipa, Macao, China

**Keywords:** Traditional Chinese medicine, Portuguese-speaking countries, Challenges, Platform role of Macau

## Abstract

With the increasing demand for traditional Chinese medicine (TCM) in Portuguese-speaking countries (PSC), local regulatory systems and relevant legislation are still insufficient and lagging, even blank in some of them. This kind of unbalanced pace either makes users of TCM exposed in potential risk or eventually obstructs the long-term development of TCM in PSC. Despite existing tremendous studies on the internationalization of TCM, there are few studies specific to PSC. Thus, by a comprehensive desk review and typical case study, this article aims to summarize current situation of TCM in PSC by a cross-regional comparison, to identify various critical challenges, and further to provide an insightful reference to impel the development of TCM in PSC.

## Introduction

The use of traditional and complementary medicine (T&CM) is found in almost every country around the world, and the demand for its services continues to grow [1, 2]. Traditional Chinese medicine (TCM) includes various forms of acupuncture, herbal medicine, Qigong and dietary therapy, and contributes to addressing some challenging diseases, especially in the area of non-communicable chronic diseases and population aging [1, 3]. It has existed for thousands of years in China, practiced with conventional medicine at each level of the healthcare service and covered by both public and private insurance [1, 2]. In recent years, Chinese government has identified TCM as a priority, and reflected a determination to the internationalization of TCM [4].

Meanwhile, featured with its accessibility and affordability, TCM has gained increasing interest and acceptance as a form of T&CM practices worldwide [2, 5].The World Health Organization (WHO) develops and enforces policies, regulations and guidelines for the development of T&CM, including TCM, which is also a major step for TCM internationalization and a help in setting up TCM centers around the world [1, 2, 6, 7]. In 2004, the WHO Traditional Medicine Strategy 2014–2023 made clear that the establishment of regulatory systems should cover not only the products, but also extend to the aspects of T&CM practices and practitioners, further ensuring the safety and quality of T&CM practices [2].

**Fig. 1:**
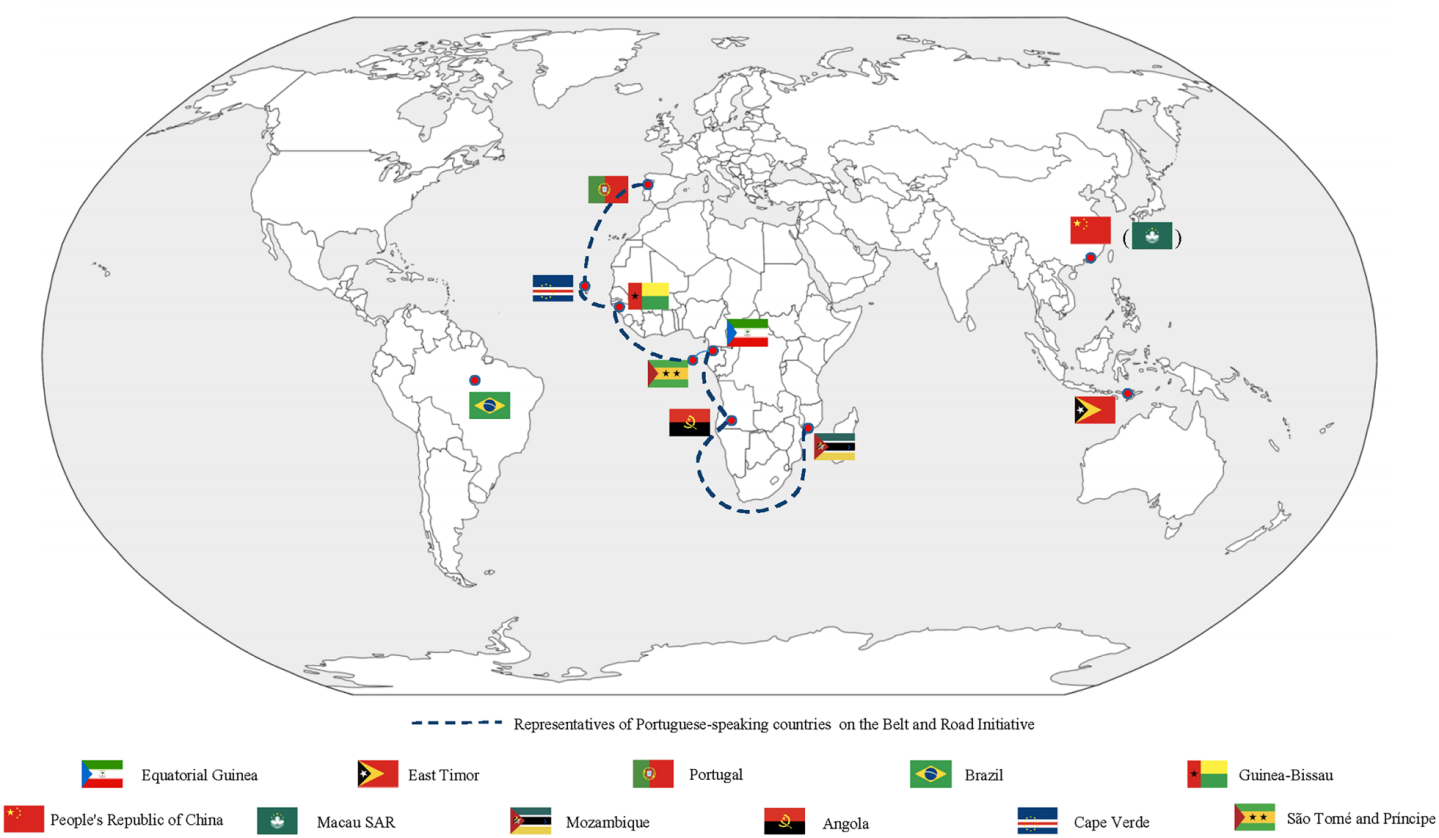
Geographical distribution of Portuguese speaking countries

TCM has witnessed rapid development in Portuguese-speaking countries (PSC) with the continuous implementation of the Belt and Road Initiative (BRI). The community of PSC is an international organization and political association across four continents, and consists of nine countries that are bound by language due to the history of the colonization process [7]. The member states are Portugal, Brazil, Timor-Leste, Angola, Cape Verde, Equatorial Guinea, Guinea-Bissau, Mozambique, and Sao Tome and Principe, and they are well-positioned to make great progress as participants in China’s promising BRI (See Fig. 1) [8]. Hence, TCM is embracing unprecedented opportunities for the development in PSC. In addition, a significant amount of attention has been given to China’s relationships with PSC. At least 2 billion yuan in aid is given to help prevent and control malaria and conduct research on traditional medicine in PSC [9]. And the efficacy and affordablility of TCM brought by Chinese medical teams arouses the interest of local people in these countries [10]. At present, TCM has been legalized by Brazil and Portugal, and other PSC are in the process of developing the regulation for T&CM [1, 7, 8, 11]. Notably, the “Mozambique model” of TCM development, with its emphasis on “promoting medicine through practice” with drug rgistration has a great appeal to many in the Portuguese-speaking world, particularly its African members.

However, there are still many challenges standing in the way of promoting TCM development in PSC, and limited studies are focus on the international development of TCM in PSC. Thus, by a comprehensive desk review and typical case study, this article aims to summarize current situation of TCM in PSC by highlighting regional similarities and differences, to identify relevant critical challenges from different aspects, and further to provide an insightful reference to impel the development of TCM in PSC.

## Developmental background of TCM in PSC

In PSC, TCM is termed a form of T&CM practices and commonly used in each nation [1]. Given the increasing consumer demand and market value for TCM, Brazil and Portugal have built up legislation and regulation on TCM to safeguard the public while using TCM practices and products [11, 12]. On the contrary, there is no regulation regarding TCM in other PSC but different progress in establishing the regulation for T&CM [1, 7].

At the request of the World Health Assembly (WHA) in 2009, and in line with the WHO Traditional Medicine Strategy 2002–2005 and the WHO Traditional Medicine Strategy 2014–2023, most PSC have taken steps to promote the safety, quality and efficacy of T&CM by developing national policies, regulatory frameworks [2, 5]. In fact, the advocacy of integrating T&CM into mainstream global health care conduces to promoting TCM into overseas market, especially for countries with experience of using TCM as complementary medicine. In addition, legislation for TCM in both Brazil and Portugal is based on the guidelines and strategies provided by WHO for T&CM development and enforcement. Thus, understanding the current situation of T&CM development in PSC is necessary and contributes to legislating TCM in these nations. Table 1 is about the status of T&CM development in PSC, including national policy, law or regulations, office, license or certificate, and education. The data is collected from WHO Global Report on Traditional and Complementary Medicine 2019 and official websites in these countries [1, 13, 14].

**Table 1:** The status of T&CM development in PSC

	Brazil	Portugal	Mozambique	Equatorial Guinea	Guinea-Bissau	Angola	Cape Verde	Sao Tome and Principe	Timor- Leste
National policy	National Policy for Integrative and Complementary Practices (PNPIC)	/	Traditional Medicine Policy and Strategy to be Implemented	/	National Policy and Regulation of Guinea-Bissau Traditional Medicine	National Policy of Traditional Medicine Complementary to the Health System	/	/	National Drugs and Medicines Policy
National office	National Coordination Office on Integrative and Complementary Practices	National Council of NCT	Traditional Medicine and Medicinal Plants Studies Department	Traditional Medicine Department	Directorate of Community Health Care Services and Promotion of Traditional Medicine	National Office of TM/CAM	/	/	/
Law or regulation	Ordinance GM/MS No.971	Act 71/2013	/	/	/	/	Deliberacao No 06/2016	/	/
Regulatory authority	National Health Surveillance Agency (ANIVISA)	National Authority of Medicines and Health Products (INFARMED)	Ministry of Health (MISAU)	/	/	/	Ministry of Health	/	National Drug Administration
License or certificate	A T&CM license or certificate is required for practice, with self-regulation by delegated special technical associations	T&CM professionals are required to hold a license issued by Central Administration of the Health System (ACSS)	City and community governments issue the license required for T&CM practice	A T&CM license or certificate is required for practice, issued by the national Government	Regulation of T&CM providers is enforced at national, province, city and community levels	/	/	/	/
Education	University level T&CM training programs	University level	University level T&CM training programs	T&CM training programs	/	/	/	/	/
Types of T&CM practices	Commonly used T&CM practices	Commonly used T&CM practices	Commonly used T&CM practices	Commonly used T&CM practices	Commonly used T&CM practices	Commonly used T&CM practices	Commonly used T&CM practices	Commonly used T&CM practices	/

Commonly used T&CM practices: indigenous TM practices, acupuncture, homeopathy, ayurvedic medicine, chiropractic, herbal medicines, naturopathy, osteopathy, traditional Chinese medicine, and Unani medicine

### Wide use of T&CM in PSC

From the table, of the total nine nations in PSC, significant progress was seen in the development of national policies, national offices, and regulatory systems for T&CM. Six nations (Portugal, Brazil, Mozambique, Equatorial Guinea, Guinea-Bissau, and Angola) reported the presence of a national office in charge of issues related to T&CM, and five (Brazil, Mozambique, Guinea-Bissau, Angola, and Timor-Leste) had a national policy on T&CM. Furthermore, they have realized the importance of regulation on T&CM practitioners, reflecting in the aspects of license and education requirements. Four nations (Portugal, Brazil, Mozambique, and Equatorial Guinea) explicitly required that T&CM practitioners should receive trainings and hold licenses for practice. It is also worth mentioning that TCM is commonly used in most PSC.

### Different development progress in PSC

At present, since different economic levels, health system structures and national priorities, Portuguese-speaking African countries (PSAC) and Timor-Leste obviously lagged behind Brazil and Portugal for all indicators in terms of T&CM governance[1, 2]. Both countries have been concerned with the integration of T&CM into their healthcare systems. They have comparatively well-established regulatory systems and education systems for T&CM products, practices and practitioners [1, 15, 8, 16]. In Portugal, for instance, Central Administration of the Health System (ACSS) is responsible for the registration of T&CM practitioners, and National Authority of Medicines and Health Products (INFARMED) is in charge of evaluating, authorizing, regulating and controlling human medicines as well as health products [12, 16]. Whereas PSAC with long history of traditional medicine use are at a slow pace in the development of regulatory systems for T&CM. From the time of issuing national polices till now, these nations are still faced with the absence of law and regulation for T&CM, the deficient T&CM research infrastructure, and the insufficient regulation of T&CM products and practitioners [1, 17, 18].

The process of T&CM development varies even in PSAC where 80 % of African population depends on T&CM [19]. Of the total six nations in PSAC, Mozambique is showing commendable advance. Mozambique has founded two institutes (Traditional Medicine Institute and Ethnobotanical Development Centre) and expert committee for the purpose of legislating and regulating T&CM since Mozambique issued national policy in 2009 [1, 20]. As of 2017, national laws and regulations on T&CM are being developed. Similar to Equatorial Guinea, T&CM practitioners are required to hold licenses before practicing T&CM [1]. So far, 110,000 medical practitioners have been registered in Mozambique through various training courses [20]. As for T&CM education, Mozambique cooperates with foreign universities for the training of masters[1, 21]. In 2016, a master’s degree in natural products was provided by the Universidade Pedagogica in collaboration with Lisbon Pharmacy University [1]. In contrast, other nations in PSAC lag behind in the legislation for T&CM, especially Timor-Lester, Cape Verde, and Sao Tome and Principe. All of them lack mechanisms to monitor the safety of T&CM practices, and lack of education and training for T&CM practitioners [1, 7].

## Analysis of representative countries on TCM legislation

In recognition of the fact that the increasing demand for TCM in PSC, policies and strategies must be formulated by national health authorities to ensure the safety and quality of it. Accordingly, it may be useful to consider the experience and information of Brazil and Portugal when other nations in PSC develop and implement national policies and TCM regulations. Table 2 summarizes the practice and regulation of TCM in both Brazil and Portugal.

**Table 2:** Practice and regulation of TCM in Brazil and Portugal

	National Policy	Regulation on TCM	Regulatory authority for TCM practitioners	Regulation on TCM practitioners	License or certificate	Education	Health insurance
Brazil	National Policy for Integrative and Complementary Practices (PNPIC) [23]	RDC 21/2014: regulate TCM products [25] RDC 26/2014: registration of herbal medicine [24]	Regional councils [26]	Ordinance GM/MS No.971: establish the health professionals qualified to use TCM therapy within SUS [23]	TCM license or certificate is required for practice, registered with regional councils [26]	University level [29] Professional training [30]	Covered by both public and private health insurance [1]
Portugal		Law nº45/2003: establish the legal framework for acupuncture [32] Law nº71/2013: establish the legal framework for TCM [34]	Central Administration of the Health System (ACSS) [40]	Law nº71/2013: establish the emission of professional licenses to practitioners [34] Ordinance 207-G/2014: establish and characterize the functional content for the degree of TCM Specialist [35] Ordinance 207-F/2014: establish and characterize the functional content for the degree of acupuncturist [36] Ordinance 181/2014: establish the time period and criteria for practitioners to apply for professional license [39]	Undergraduate level degree [37, 38] Transitory system for existing non-university level practitioners [39]	Ordinance 172-C/2015: establish legal requirements to be satisfied by the study cycle for Acupuncture degree [37] Ordinance 45/2018: establish legal requirements to be satisfied by the study cycle for TCM Specialist degree [38]	Covered partially by some private health insurance [1]

### Brazil

TCM was introduced into Brazil in the twentieth century and regulated in 1995, when acupuncture was recognized as a medical specialty by the Federal Council of Medicine (CFM) [22]. Then in 2006, the National Policy for Integrative and Complementary Practices (PNPIC) was launched to incorporate TCM/acupuncture, anthroposophical medicine, homeopathy and social thermalism into the Brazilian Unified Health System (SUS), thus TCM treatment officially entered the public hospitals in Brazil and was reimbursed by both public and private health insurance [1, 23]. In 2014, National Health Surveillance Agency (ANIVISA) issued two resolutions related to TCM, they are RDC 21/2014 and RDC 26/2014. The former deals with the production and marketing of TCM products, while the latter deals with the registration of herbal medicines [24, 25].

Professional activities are regulated by Brazilian federal councils, which stipulate all professionals must be registered with regional councils [26]. The Federal Council of Physiotherapy and Occupational Therapy (COFFITO) is the first professional board to recognize acupuncture as an occupational practice through Resolution No. 60/85 [27]. Since then, physical therapists are legally qualified to practice acupuncture as part of their profession. In addition to physical therapists, other medical doctors and allied health professionals within SUS are qualified to use TCM therapy according to Ordinance GM/MS No.971 [23]. In 2016, more than 2 million T&CM professionals were registered in the Basic Health Units of SUS, of which 770,000 were from TCM/acupuncture [28].

In Brazil, there are many free courses on TCM provided by private institutions, such as Chinese diet therapy, herbal medicine [29]. The development of TCM education is mainly reflected in the research of acupuncture. Early in 2002, the Ministry of Education regulated courses on the acupuncture therapy, which were organized and taught by medical doctors, and now there are over 8500 acupuncturists in Brazil [30]. On the other hand, Brazil is the only country outside China that has created a residency program in acupuncture, consisting of 5760 training hours [22]. Furthermore, the federal government has made great effort in the scientific development, including TCM research. It has invested US$ 10 billion in the development of education and established a network of 354 universities and institutes for teaching student’s science and technology and training new professors and researchers [31].

### Portugal

Portugal is the first European country acknowledging TCM profession. The integration of acupuncture into the Portuguese healthcare system, along with another five Complementary and Alternative Medicines (CAM, which called T&CM in this paper) therapies, was started with a legal document (Law nº45/2003) from 2003, although its practice goes further back to the 1970 s [32, 33]. After 10 years in September 2013, Law nº71/2013 was issued by Portuguese government to regulate the professional exercise and application of the six T&CM therapies and add TCM therapy. Also, the new law establishes and approves the emission of professional licenses to practitioners, stipulating the seven therapies can only be practiced by practitioners with bachelor’s degree and a public registered professional license [34].

In order for this new law to be fully implemented, some specific regulations have been published over the next few years. Of which Ordinance 207-G/2014 and Ordinance 207-F/2014 legally defined the duties of and outline practice standards for TCM specialists and acupuncturists, respectively [35, 36]. It wasn’t until 2015 and 2018 that the contents of higher education degrees for acupuncture and TCM professionals would be defined legally, respectively [37, 38]. Their access requirements and study cycles were specified, each at least to undergraduate level. According to Ordinance 181/2014, a special transitory system would be applied for those professionals who have already been working in the market prior to the publication of Law nº71/2013 [39].

The regulatory authority for T&CM professionals is the Central Administration of the Health System (Administração Central do Sistema de Saúde—ACSS), which provides a permanent online registry and publicly online consultation for all T&CM professionals. So far, ACSS has issued professional licenses for 57 TCM specialists and 1246 acupuncturists [40]. In addition, courses on TCM and acupuncture has already been built up in six Portuguese universities [41]. Of which, Porto University has established the first Master of TCM degree for medical doctors. It contains four semesters with a total of 3600 study hours, and various TCM disciplines including acupuncture, Tui Na (massage therapy), Qi Gong (energy gymnastics), phytotherapy (therapy using plants) and Diet therapy (food therapy) [42].

As for the health insurance, the fees for acupuncture and prescribed TCM treatment are partially covered by some private health insurance companies. Some insurances have T&CM practitioners in their health staff who can use the services of these insurances. However, the services of external practitioners of T&CM cannot be reimbursed [1].

The current process for legislating profession of TCM, including acupuncture, in Brazil and Portugal guarantees safety for both people using these services and practitioners providing the services, which is also a huge step not only for TCM development but also for all T&CM development in general. Furthermore, the valid legislative strategy between these two countries gradually generates a domino effect, leading more nations in PSC legislating TCM and thereby protecting their citizens.

## Challenges of TCM development in PSC

Along with the changes in medical models and public health perception, TCM is commonly used in PSC as a complementary medicine, and gains increasingly attention from national health authorities in PSC. However, a multitude of challenges and difficulties need to be overcome.

### Quality and efficacy

Expanding the credibility of TCM mainly depends on developing an evidence base for quality and efficacy. The establishment of controllable quality standard is a national strategy used in the internationalization of TCM [43]. This is not only a challenge for the development of TCM in PSC, but also a major problem facing the internationalization of TCM [44].

The continuous growth and globalization of the Chinese medicine market cannot prevent people from challenging its efficacy and scientific background. Though, topics of TCM system were discussed in some journals, its efficacy has not been well explained [45, 46, 47]. The chemical composition of TCM is very complex with the result that its biological effects are from the interaction of many active ingredients, even with so-called inactive ingredients [48]. However, given the limitations of science and technology, personnel and materials, previous research on TCM is focus on the single ingredient, while it cannot represent the full efficacy [48]. According to Chinese Pharmacopoeia (2020), a total collection contains 2711 types of herbs, and varieties that have undergone systematic chemical composition studies do not exceed 20 %. This phenomenon is one of the reasons why TCM is difficult to be accepted in foreign countries [49].

Furthermore, the quality of TCM is also affected by its variety and origin, such as its harvest time and processing methods [50]. European countries have strengthened the detection of metals, pesticide residues and microbial content in TCM, and incidents of excessively high concentrations of harmful substances in some TCM have been widely reported. The spread of negative news of TCM quality is not conducive to the development of TCM in PSC [51]. The effective risk assessment method for traditional Chinese medicine has not been developed in China, and the guidelines for heavy metal safety standards have not been perfect [52]. Thus, the adoption of genuine standards should be established to protect the resources of TCM materials, and regulate the competition in the medicinal materials industry [50].

It is worth noting that the ethnic groups in PSC are different from Asian countries, so it is necessary to set up complete clinical data to support the effectiveness of TCM in PSC. Requirements of TCM recorded in different national pharmacopoeias and regional monographs varied a lot. There have been only limited efforts at parallel development of international standards and methods, and existing international standards such as ISO/TC 249 are still under development [53]. Lianhua Qingwen Capsule is registered in six countries after COVID-19: Canada, Brazil, Romania, Thailand, Indonesia, and Mozambique, under various labels such as Chinese patent drug, natural health food, or food supplement. Among the above countries, Brazil and Mozambique are state members of PSC. Compared with failing to register in other places such as Europe and the United States, the access conditions for the effectiveness and quality of TCM is more relaxed in PSC [54]. Only registration approval Lianhua Qingwen Capsule has been obtained, and there is no more evidence to show that large-scale sales are carried out in these countries.

### Policy and regulation

Lacking legal mechanisms and regulations is another formidable challenge for TCM to penetrate the PSC’s market. Despite the use of acupuncture and TCM products is having impressive consequences in many countries, but the legitimacy of TCM has not yet been completely recognized [55]. In PSC, the development of TCM is unstable due to the varying degrees of acceptance among policy makers and public.

Only Brazil and Portugal have legislation related to TCM, while other PSC have not legalized TCM [24, 38] However, legal frameworks are with unclear definitions of responsibilities, resulting in regulatory gaps and overlaps. Chinese herbal medicine and acupuncture are two inseparable treatment methods within the theoretical framework of TCM, but they are considered as two independent treatment methods in some PSC. For acupuncture and moxibustion, Brazil has detailed laws and regulations, stipulating the education, qualification, and supervision of practitioners [26]. Until 2014, TCM was begun to register as a drug through RDC 21 / 2014 by ANIVSA but there were no detailed regulations on the registration process and subsequent supervision [24]. Without relevant regulations, TCM is practiced with little government oversight and without patient or consumer protection. Therefore, national policies should encompass legislation and regulation of practice and products; education, training, and licensing of providers; and research and development.

In Portugal, only acupuncture has been legalized, while there are no special regulations for TCM registration and regulation [37, 38]. For parallel imports, there is a legal presumption that a medicine subject to EU parallel trade will have the same qualitative and quantitative composition, pharmaceutical form, and indications. Brazil also has the same regulations, which can refer to the EU standards [38]. Taking the EU as an example, drugs exported to the EU market need to meet the EU’s “Good Manufacturing Practices for Pharmaceutical Manufacturing” (GMP) review. The quality of drugs must meet the quality standards of GMP, and traditional botanicals must follow the EU Traditional Plant Drug Registration Procedure Directive. It needs to comply with the registration procedures and technical standards of the directive [55]. However, the medical theoretical systems and standards between Chinese and orthodox medicine are very different, which makes it difficult for Chinese medicine products to meet the above-mentioned review standards, so it is extremely difficult for medicines to go on the market.

Moreover, it is difficult to measure TCM scientifically and assess them accurately, because the clinical efficacy of TCM diagnosis and treatment techniques has uncertainties [56]. The lack of high-quality clinical research on TCM in PSC especially randomized controlled trials (RCTs) based on TCM symptom measurement indicators. PSC in African has no clinical research organizations and bioequivalence centers [57].

### Access conditions

Due to the colonial ties, the United States and EU have dominated the supply of medicines to PSC’s market, and taken steps to enhance its access threshold, which makes China harder for promoting TCM into PSC. For example, Chinese researchers discovered the derivatives of Artemisinin have the effectiveness against malaria, but the marketing rights of its derivatives, Compound Artemether was bought by Novartis in 1994 and then obtained global patent authorization in 1999. Beijing Academy of Military Medical Sciences was unable to develop overseas markets due to many difficulties at that time, so it transferred global patents to Novartis [58]. To avoid similar incidents, China has to take advantage of low price of TCM, and adopt price competition strategy. The drug pricing in China is one third of that in other drug supply countries, of which the overall price index represents 35 % of the average level of developed market, and 55 % of the pharmerging market [59]. Moreover, the characteristics of the pharmaceutical market of PSC are aggravating the fierce competition.

Another challenge is lacking robust and continuous supply chain in most PSC. Most PSC are lack of good infrastructure such as transportation systems, communication networks and constant power supply [57]. Lacking distribution severely limits the accessibility of medicines from manufacturing sites to patients. Medicine can be either distributed by the importers or via the manufactures’ distribution channels to hospitals and pharmacies, and they are highly chaotic and unregulated [60]. Even if the drug is tap proved, the lack of drug access will have a negative impact [61]. Too much investment in the field of circulation will also increase costs. It is clear from the forgoing that access to TCM in PSC is very limited.

### Cultural divergence

Cultural divergence is the primary factor restricting the popularity of TCM in PSC. TCM include practices and procedures such as acupuncture, manual therapies and herbal medicine comprised of some of botanical ingredients together in a formula [62]. TCM emphasizes on the integrity of the human body and the integral relationship between human and its natural and social environment [63]. PSC are affected by the history, and most medical development rely heavily on orthodox medicine [2]. Although PSC have the habit of using complementary and alternative medicine, TCM is still regarded as a profession that requires additional learning [22]. The theory of TCM, such as Qi and meridians, presents some challenges to its popularization in PSC [64]. Only some universities in Portugal, Brazil, and Mozambique offer relevant courses [10]. The cultural differences directly lead to few practitioners who choose to engage in the upstream and downstream industries of TCM, and TCM has not found general public acceptance from patients.

Furthermore, the cultural obstacles to promoting TCM internationalization can result from the linguistic diversity between patients and TCM practitioners. Common TCM treatments include herbal medicines, acupuncture, massage, and food therapy. At present, TCM medicines are only approved in countries such as Mozambique, and large-scale use and production are still not possible. Only acupuncture and moxibustion are provided in countries such as Portugal, Brazil, and Mozambique [10]. Doctors engaged in acupuncture and moxibustion are currently mainly medical teams dispatched by China in PSC, and the limited Portuguese proficiency may reduce the accuracy of diagnosis or treatment [10]. Complicating matters further, the professional terms and metaphors of TCM are difficult to navigate even for patients or TCM practitioners with high language competence. The lack of Portuguese translators also hindered the process and promotion of related technologies in PSC.

## Insights from a real-world case in the exploring way

Promoting the internationalization of TCM in PSC is a process of continuous exploration. Although there have been a large number of studies exploring the development strategies and paths in the internationalization of TCM [44, 65, 66], most of these studies are based on theoretical research. Therefore, the few successful cases of legalized TCM products entering the international market need to be further demonstrated in combination with actual cases.

Due to historical and linguistic ties, Macau has close and extensive connections with PSC, and is well positioned in creating a favorable environment for TCM promotion. On one hand, Macau has legal system based on those of Portugal and follows the legal regime similar to other PSC, which helps people understand the respective legal systems between China and PSC [67]. On the other hand, the conjunctive use of TCM and western medicines is common within the Macau public. The government has integrated TCM into its public health care system since 1999, and is promulgating legislation regarding the registration of TCM to ensure the quality and safety of TCM manufacture, import and distribution in Macau [68].

In addition, Macau seizes the opportunitie of the BRI to drive TCM into the international community. BRI has a specific branch dedicated to facilitating communication among countries in order to create a platform for health services and the health industry, develop international assistance, and facilitate personnnel trainings and medical research. For instance, the establishment of the WHO Collaborating Centre for Traditional Medicine in Macao and the Traditional Chinese Medicine Science and Technology Industrial Park of Co-operation between Guangdong and Macau (GMTCM Park) aims to facilitate the moderate diversification of Macau’s economy and the internationalization of TCM [69, 70].

For promoting TCM towards PSC, Macau has adopted a unique development model based on the characteristics of different nations in PSC, including their distinctive national policies and regulations, registration system, regulatory system, practice standards, market size, and public acceptance of TCM. Taking Mozambique as an example, with a medium-sized population and a relatively large market, the African republic has a high public recognition for traditional medicine products. According to the 2015 data of the National Institute of Statistics of Mozambique, there were 1435 medical institutions including 64 hospitals, 1307 polyclinics, 164 public clinics, 20,826 hospital beds, 1042 doctors and 5213 nurses, mainly utilized by the masses under Mozambique’s free health care system, or high-income groups covered by medical insurance [71]. The platform role of Macau has been fully leveraged to pushed for the international registration and trade of TCM through incorporating a collaborative model of “promoting medicine through practice” with drug registration. So far, six TCM products has been successfully registered in Mozambique, including Cheong Kun Pain Reliever Oil of Cheong Kun Oil Factory, Lavender Healing Oil and Lotus Cream of Macau-Union Pharmaceutical, Lianhua Qingwen FluGone Capsules of Yiling Pharmaceutical, Huoxiang Zhengqi Gastrointestinal Serum of Taiji Group, and Essential Balm of Narcissus Pharmaceutical. Among them, the Cheong Kun Pain Reliever Oil and Lianhua Qingwen FluGone Capsules have been imported and sold in Mozambique to good reviews, with the latter receiving positive market effects in the prevention and control of COVID-19 in 2020 [72, 73, 74, 75]. As for personnel training, ten TCM professional training programs have been organized for doctors and physiotherapists from the Mozambican Ministry of Health. And the programs have trained about 209 public doctors, physiotherapists and pharmacists, and treated more than 7000 Mozambican patients using TCM therapies [76]. Furthermore, TCM Center has been founded in Mozambique’s capital city, Maputo with the support of GMTCM Park in 2018. This center is a key platform for transmission of TCM culture, provision of TCM international training, and promotion of TCM product application, registration and trade [10].

As a practical case study in the exploration of development paths and strategies in the internationalization of TCM, Macau has succeeded in promoting the popularization and application of TCM in Mozambique, and initiating a new model for product registration and subsequent market channel expansion through the international exchanges and cooperation work. The “Mozambique model” has also been replicated and promoted in other PSAC such as Angola, Cape Verde and Guinea-Bissau [76, 77].

The development of TCM in PSC should be adapted to local conditions, taking into account the national conditions of different countries. For Portugal and Brazil, which already have a legal framework, further improvements should be made under the existing framework. There are regulations for health professionals, but there is still no legal basis for special regulations for relevant practitioners, pharmacists and other related personnel, and there are no clear regulations on the qualifications and responsibilities of health professionals. The establishment of laws and regulations needs reference to the principle of personnel admission and registration specialization in Macau. China should make full use of Macau’s platform role and strengthen cooperation with Portuguese-speaking countries to achieve mutual benefit and win-win results. For other PSC that have not yet enacted legislation, legislation should be promoted from the government level, for example, to establish a legal system with reference to the legal framework of Macau. Collaboration between cultural organizations should be increased, using channels like GMTCM park to establish the theoretical establishment and cultural background propaganda of the current medical industry practitioners on TCM, and strengthening the professional knowledge exchange salon, practical guidance and clinical training between China and PSC. Meanwhile, a training mechanism for institutions of higher learning should be established. If TCM can be further officially recognized in Mozambique and other nations in PSC, it will produce a positive influence on other countries.

## Conclusions

In conclusion, regulatory systems and legislation of TCM are lagging in most PSC, except that Brazil and Portugal have had relevant legislations related to TCM. Moreover, different PSC have significant differences in regulatory and legislative foundation on TCM. Critical challenges of the development of TCM in PSC include quality and efficacy, policy and regulation, access condition, and cultural divergence. Macau can serve as a platform to promote TCM towards PSC, in terms of its bridging role in connecting China with Portugal historically and politically, established social basis of coexistence between the Chinese and Western cultures, and emerging scientific and technological achievements on TCM.

## Data Availability

Not applicable.
